# Tick-borne pathogens in ticks collected from dogs, Latvia, 2011–2016

**DOI:** 10.1186/s12917-019-2149-5

**Published:** 2019-11-06

**Authors:** Agne Namina, Valentina Capligina, Maija Seleznova, Rudolfs Krumins, Darja Aleinikova, Agnija Kivrane, Sarmite Akopjana, Marija Lazovska, Inese Berzina, Renate Ranka

**Affiliations:** 1 Latvian Biomedical Research and Study Centre, Ratsupites Str. 1, Riga, LV-1067 Latvia; 2 Latvia University of Agriculture, Jelgava, Latvia

**Keywords:** Tick-borne pathogens, Ixodes, Dermacentor, Ticks, Dogs, Latvia

## Abstract

**Background:**

Different tick species are able to transmit different pathogens, and tick-borne diseases are of substantial concern worldwide for both humans and animals. Environmental changes and changes in the range of tick species, including *Dermacentor reticulatus* in Europe, can affect the spread of zoonotic pathogens. The aim of this study was to investigate the prevalence of the tick-borne pathogens in ticks removed from dogs in Latvia, and to explore possible changes between years 2011 and 2016.

**Results:**

In 2011, only *Ixodes* ticks (221 *Ixodes ricinus* and 22 *Ixodes persulcatus*) were collected from dogs, while in 2016 tick samples belonged to *Ixodes ricinus* (360), *Ixodes persulcatus* (2) and *Dermacentor reticulatus* (27) species. In total, 35.8 and 40.0% of adult ticks were pathogen-positive in 2011 and 2016, respectively; the difference was not statistically significant (*P* > 0.05). The molecular analysis indicated the presence of 13 tick-borne microorganisms; the most prevalent pathogen was *Rickettsia*, followed by *Borrelia burgdorferi* sensu lato group spirochetes, *Anaplasma phagocytophilum* and *Babesia* species. *Borrelia miyamotoi* was also present. A co-infection with two and three tick-borne pathogens was detected in 7.9 and 7.4% of *Ixodes ricinus* and *Dermacentor reticulatus*, respectively. The results of this study confirmed that the spread of novel vectors could bring additional risk of exposure to novel emerging pathogens to pets and their owners, as both *Babesia canis* and *Rickettsia raoultii* were shown to be highly associated with *Dermacentor reticulatus* ticks in Latvia.

**Conclusions:**

This study demonstrates the potential danger from the inadvertent introduction of novel disease pathogens and vectors. Awareness of co-infections and *Dermacentor reticulatus*-related pathogens needs to be increased.

## Background

Ticks are able to transmit numerous disease agents such as viruses, bacteria and protozoa, and tick-borne diseases are of substantial concern worldwide for both humans and animals. Different tick species are able to transmit different diseases, and vector competence depends on genetic determinants affecting the ability of a vector to transmit a pathogen [1]. The widespread European tick species *Ixodes ricinus* (*I. ricinus*) acts as a vector for a large variety of pathogens of medical and veterinary importance including *Borrelia burgdorferi* sensu lato (*B. burgdorferi* s.l.), tick-borne encephalitis virus, *Anaplasma phagocytophilum* (*A. phagocytophilum*), *Francisella tularensis*, *Rickettsia helvetica* (*R. helvetica*), *R. monacensis*, *Babesia divergens* (*B. divergens*) and *Babesia microti* (*B. microti*), while the meadow or ornate dog tick *Dermacentor reticulatus* (*D. reticulatus*) is an important vector for *R. raoultii*, *R. slovaca*, *Babesia canis* (*B. canis*), *Babesia caballi*, *Theileria equi*, *Anaplasma marginale*, and the brown dog tick *Rhipicephalus sanguineus* - for *Babesia vogeli* and *Rickettsiales* in the Mediterranean region [2–5]. The taiga tick *Ixodes persulcatus* (*I. persulcatus*), which is found in eastern Europe, northern Asia and, recently, has expanded to Finland and northern Sweden, transmits a wide range of human and animal pathogens including tick-borne encephalitis virus, *B. burgdorferi* s.l., *Ehrlichia muris*, *B. microti* and *A. phagocytophilum* [6, 7].

Dogs are hosts of several species of ticks, and surveillance of ticks and tick-borne pathogens is undoubtedly important in order to monitor the distribution of both vectors and tick-borne diseases. In addition, it helps to raise awareness of tick-borne diseases in dog owners, who could be ignorant of the tick-borne pathogen-associated risks to their pets. On the other hand, the recent study has showed, that pet owners, whether of cats or dogs, are at increased risk of developing tick-borne disease [8]. Subclinically infected companion animals could provide a reservoir for human tick-transmitted infectious agents, and the importance of a One Health approach was emphasized, calling physicians and veterinarians to unify their efforts in the management of tick-borne zoonoses [9, 10].

Tick-borne diseases in Latvia, a Baltic state in Northern Europe, has been a major human health concern for many years and in the last decade has gained importance in the veterinary field as well. Up until last decade only two epidemiologically-important tick species – *I. ricinus* and *I. persulcatus* – were present in Latvia, but the appearance and spread of *D. reticulatus* populations and reported local clinical cases of canine babesiosis have raised concerns over the risks to pets posed by vector-borne diseases [11, 12]. The aim of this study was to investigate the prevalence of the tick-borne pathogen species in ticks removed from dogs in Latvia, and to explore possible changes between years 2011 and 2016.

## Results

**Fig. 1:**
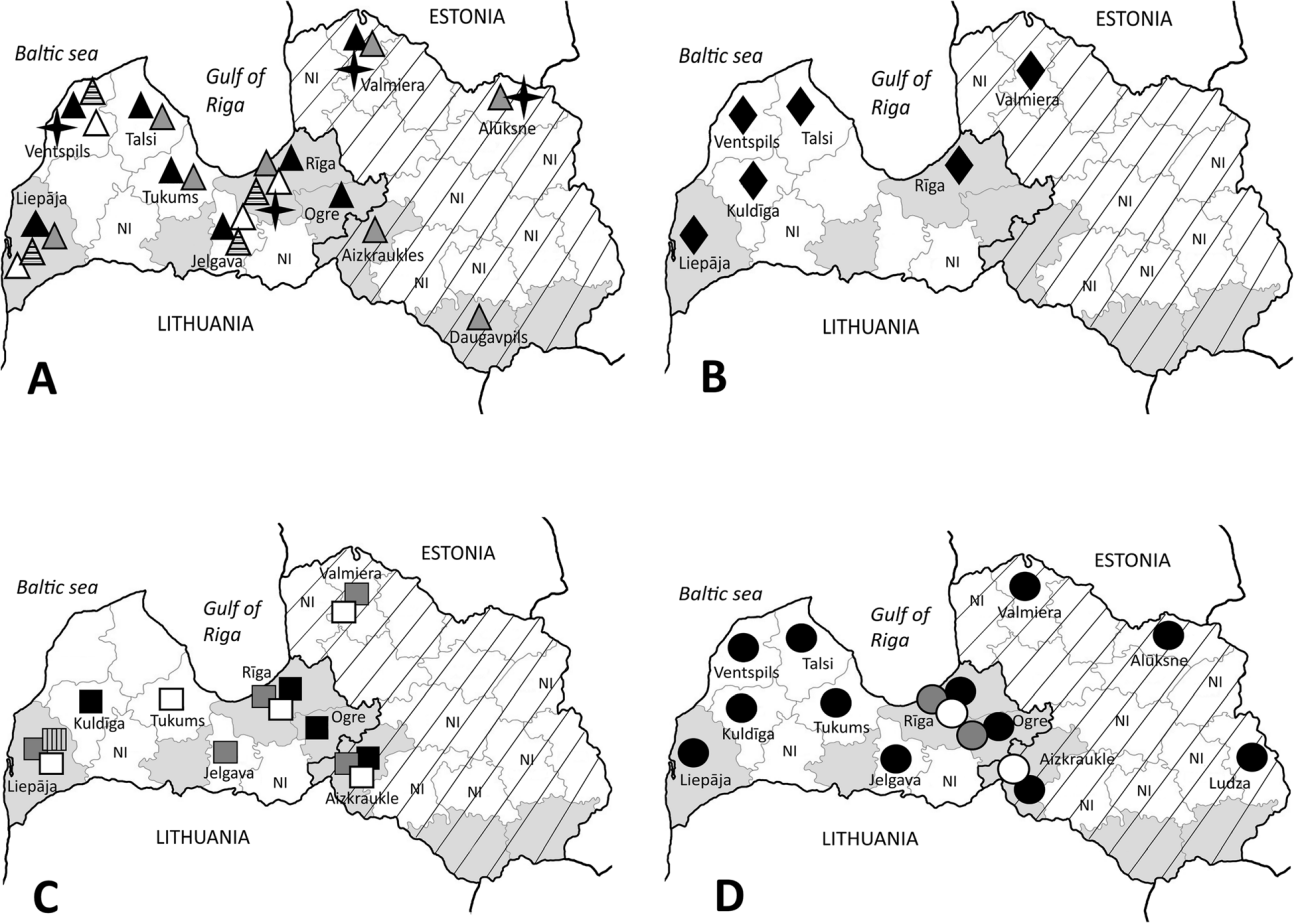
Tick sampling sites and tick-borne pathogen species in Latvia. The name is provided only for regions where positive samples were obtained. NI: the region was not included in the study. The sympatric area for *Ixodes persulcatus* and *Ixodes ricinus* tick species according to Karelis et al. (2012) [13] is highlighted by diagonal stripes. The regions where *Dermacentor reticulatus* tick species were obtained is highlighted in grey. **a** *Borrelia* genospecies: four-point star *B. miyamotoi*, white triangle *B. valaisiana*, black triangle *B. afzelii*, gray triangle *B. garinii*, striped triangle *B. spielmanii*. **b** *Anaplasma phagocytophilum*. **c**: *Babesia* genospecies: black rectangle *B. canis*, gray rectangle *B. microti*, white rectangle *B. venatorum*, striped rectangle *B. capreoli*. **d**: *Rickettsia* genospecies: black circle *R. helvetica*; gray circle *R. monacensis*, white circle *R. raoultii*. The map drawing is owned by the Authors

Overall, 632 adult ticks from dogs were analyzed (Table 1). The mean intensity of the infestation in animals was 2.76 (243 ticks/88 dogs; median = 1; range: 1–32 ticks) and 2.08 (389 ticks/187 dogs; median = 1; range: 1–42 ticks) in years 2011 and 2016, respectively; this difference was not statistically significant (*P* value = 0.0609). As was expected, two *Ixodes* tick species were identified, i.e. *I. ricinus* and *I. persulcatus*. No *Dermacentor* ticks were present among 243 samples collected in year 2011, however, in 2016, almost 7% of ticks removed from dogs in different regions of Latvia were *D. reticulatus* (27/389). This increase was statistically significant (*P* < 0.0001). *D. reticulatus* ticks were obtained in western, southern and central parts of Latvia, including Rīga, Liepāja, Daugavpils, Krāslava, Aizkraukle, Ogre and Dobele regions (Fig. 1). This result indicates that sympatric populations of *D. reticulatus* and *I. ricinus* ticks, as well as *D. reticulatus*, *I. ricinus* and *I. persulcatus* ticks, exist in several regions of Latvia.

**Table 1:** Prevalence of pathogens in ticks from Latvian domestic dogs in years 2011 and 2016

	Year 2011				Year 2016				
	*I. ricinus* (95% CI)	*I. persulcatus* (95% CI)	*D. reticulatus* (95% CI)	Total (95% CI)	*I. ricinus* (95% CI)	*I. persulcatus* (95% CI)	*D. reticulatus* (95% CI)	Total (95% CI)	*P* value†
Ticks analysed	221	22	0	243	360	2	27	389	
Pathogen-positive ticks*	38.4 (32.29–45.02)	9.0 (1.34–29)	–	35.8 (30.03–42.01)	41.7 (36.69–46.82)	0 (0–70.98)	18.5 (7.72–37.16)	40.0 (35.10–44.79)	0.3144
*B. burgdorferi* s.l. group	14.0 (10.02–19.26)	0 (0–17.55)	–	12.8 (9.1–17.58)	8.6 (6.1–12)	0 (0–70.98)	0 (0–14.76)	8.0 (5.64–11.12)	0.0547
*B. miyamotoi*	0.5 (0.01–2.78)	0 (0–17.55)	–	0.4 (0.01–2.53)	1.4 (0.5–3.31)	0 (0–70.98)	0 (0–14.76)	1.3 (0.46–3.06)	0.4143
*A. phagocytophilum*	4.5 (2.37–8.23)	0 (0–17.55)	–	4.1 (2.15–7.5)	6.9 (4.71–10.09)	0 (0–70.98)	0 (0–14.76)	6.4 (4.35–9.35)	0.2835
*Babesia*	2.7 (1.11–5.93)	0 (0–17.55)	–	2.5 (1.01–5.41)	4.7 (2.92–7.48)	0 (0–70.98)	14.8 (5.3–33.1)	5.4 (3.52–8.15)	0.1044
*Rickettsia*	22.2 (17.18–28.12)	9.0 (1.34–29)	–	20.1 (16.32–26.56)	25.6 (21.32–30.31)	0 (0–70.98)	11.1 (3.03–28.88)	24.4 (20.41–28.93)	0.3335
Coinfections‡	10.0 (6.61–14.67)	0 (0–17.55)	–	9.1 (6–13.38)	6.7 (4.48–9.77)	0 (0–70.98)	7.4 (0.96–24.47)	6.7 (4.57–9.65)	0.2833

* Including coinfections

† *P* value was calculated for the total numbers, to compare the pathogen prevalence in years 2011 and 2016

‡ Including mixed *Borrelia* infections

Further, the prevalence of different pathogens in ticks was compared for years 2011 and 2016. In total, 35.8% (87/243) and 40.0% (155/389) of ticks removed from dogs were pathogen-positive in years 2011 and 2016, respectively; the difference was not statistically significant (*P* > 0.05) (Table 1). When the prevalence of different pathogens was compared for the years studied, no statistically significant differences were observed. Most frequently, *Rickettsia* sp. was detected in tick samples; in total, 20.1 and 24.4% of ticks were *Rickettsia*-positive in years 2011 and 2016, respectively. *B. burgdorferi* s.l. group spirochaetes were detected in 12.8 and 8.0%, and *A. phagocytophilum* – in 4.1 and 6.4% of tick samples in years 2011 and 2016, respectively (Table 1). The prevalence of *Babesia* was slightly higher for the year 2016 (5.4% vs 2.5%), however, again, statistical significance was not reached. Importantly, for a considerable portion of the ticks removed from dogs the presence of two or three pathogens was shown; the total coinfection rate was 9.1% (22/243) in year 2011, and 6.7% (26/389) in year 2016; again, this difference was not statistically significant (*P* > 0.05).

The molecular analysis indicated the presence of 13 tick-borne microorganisms in *I. ricinus* ticks: *Borrelia garinii*, *Borrelia afzelii*, *Borrelia valaisiana*, *Borrelia spielmanii*, *Borrelia miyamotoi*, *A. phagocytophilum*, *B. canis*, *B. microti*, *Babesia venatorum*, *Babesia capreoli*, *R. helvetica*, *R. monacensis* and *R. raoultii*. In *D. reticulatus* ticks, only two pathogens, *B. canis* and *R. raoulti*, were detected (Table 2). Among *I. persulcatus* ticks, only *R. helvetica*–positive samples were detected; the total prevalence was 8.3% (2/24) (Table 2). The geographical spread of all pathogens detected in this study is presented in the Fig. 1. Our results showed that the most prevalent *Borrelia* species infection in *I. ricinus* ticks collected from dogs was *B. afzelii* (3.6%) followed by *B. garinii* (1.7%), *B. valaisiana* (1.4%) and *B. spielmanii* (1.4%). The presence of *B. miyamotoi* DNA was detected in 1.0% (6/581), and *A. phagocytophilum* - in 6.0% (35/581) of *I. ricinus* samples.

**Table 2:** Prevalence of pathogens in ticks from domestic dogs in Latvia

Pathogen species*	Tick species			
	*I. ricinus* (95% CI)	*I. persulcatus* (95% CI)	*D. reticulatus* (95% CI)	Total (95% CI)
*Borrelia burgdorferi* s.l. group, total	10.7 (8.4–13.46)	0 (0–16.31)	0 (0–14.76)	9.8 (7.72–12.39)
*B. garinii*	1.7 (0.89–3.18)	0 (0–16.31)	0 (0–14.76)	1.6 (0.82–2.93)
*B. afzelii*	3.6 (2.35–5.49)	0 (0–16.31)	0 (0–14.76)	3.3 (2.16–5.05)
*B. valaisiana*	1.4 (0.65–2.74)	0 (0–16.31)	0 (0–14.76)	1.3 (0.6–2.52)
*B. spielmanii*	1.4 (0.65–2.74)	0 (0–16.31)	0 (0–14.76)	1.3 (0.6–2.52)
*B. burgdorferi* s. l. group, mix of two genotypes	2.6 (1.53–4.25)	0 (0–16.31)	0 (0–14.76)	2.4 (1.41–3.91)
*Borrelia miyamotoi*	1.0 (0.42–2.29)	0 (0–16.31)	0 (0–14.76)	1.0 (0.38–2.11)
*A. phagocytophilum*	6.0 (4.34–8.28)	0 (0–16.31)	0 (0–14.76)	5.5 (3.99–7.62)
*Babesia*, total	4.0 (2.62–5.90	0 (0–16.31)	14.8 (5.3–33.1)	4.3 (2.93–6.17)
*B. canis*	**1.0 (0.42–2.29) †**	0 (0–16.31)	**14.8 (5.3–33.1)**	1.6 (0.82–2.93)
*B. microti*	1.6 (0.77–2.96)	0 (0–16.31)	0 (0–14.76)	1.4 (0.71–2.73)
*B. venatorum*	1.2 (0.53–2.52)	0 (0–16.31)	0 (0–14.76)	1.1 (0.49–2.32)
*B. capreoli*	0.2 (0.01–1.07)	0 (0–16.31)	0 (0–14.76)	0.2 (0.01–0.98)
*Rickettsia*, total	24.3 (20.95–27.92)	8.3 (1.16–27.0)	11.1 (3.03–28.88)	23.1 (19.98–26.55)
*R. helvetica*	**23.6 (20.3–27.2) †**	8.3 (1.16–27.0	**0 (0–14.76) †**	22.0 (18.93–25.39)
*R. monacensis*	0.5 (0.1–1.58)	0 (0–16.31)	0 (0–14.76)	0.5 (0.09–1.46)
*R. raoultii*	**0.2 (0.01–1.07) †**	0 (0–16.31)	**11.1 (3.03–28.88) †**	0.6 (0.18–1.68)
Ticks analysed	581	24	27	632

* Including coinfections

† Prevalences with different indices which are significantly different with *P* values ≤0.05 are indicated in bold. *P* values were corrected for multiple testing by Holm correction

In total, 14.8% (4/27) of *D. reticulatus* ticks were *Babesia*-positive in our study compared with 4.0% (23/581) of *I. ricinus* samples. Among *I. ricinus*, four *Babesia* species were detected: *B. microti* (1.6%), *B. venatorum* (1.2%), *B. capreoli* (0.2%) and *B. canis* (1.0%). By contrast, all *Babesia* in *D. reticulatus* samples were *B. canis*, and the total prevalence of *B. canis* in *Dermacentor* ticks was significantly higher than in *I. ricinus* ticks (14.8% vs 1%; *P* ≤ 0.05).

Almost one quarter of all *I. ricinus* ticks removed from dogs were *Rickettsia*-positive (24.3%, 141/581); the vast majority of these samples were *R. helvetica* (23.6%, 137/581), and only few samples belonged to *R. monacensis* (0.5%, 3/581) and *R. raoultii* (0.2%, 1/581) (Table 2). Among *D. reticulatus*, 11.1% (3/27) were *Rickettsia*-positive, and *R. raoultii* was the sole species detected. When the prevalence of *Rickettsia* was compared between *I. ricinus* and *D. reticulatus* ticks, the difference was statistically significant for *R. helvetica* and *R. raoultii* species (*P* ≤ 0.05) (Table 2).

A co-infection with two and three tick-borne pathogens, including two genotypes of *B. burgdorferi* s.l. group, was detected in 7.9% (46/581) and 7.4% (2/27) of *I. ricinus* and *D. reticulatus* samples, respectively. In total, 19 different pathogen combinations were detected, and the composition of these co-infections mirrored the pathogens’ spectra as the sole co-infection found in *D. reticulatus* ticks was a combination of *B. canis* and *R. raoultii* (Table 3).

**Table 3:** Prevalence of co-infections detected in ticks from domestic dogs in Latvia

	Tick species			
	*I. ricinus* (95% CI)	*I. persulcatus* (95% CI)	*D. reticulatus* (95% CI)	Total (95% CI)
No. of samples analysed	581	24	27	632
*B. garinii* two genotypes	0.2 (0.01–1.07)	0 (0–16.31)	0 (0–14.76)	0.2 (0.01–0.98)
*B. valaisiana* two genotypes	0.2 (0.01–1.07)	0 (0–16.31)	0 (0–14.76)	0.2 (0.01–0.98)
*B. valaisiana* + *B. spielmanii*	1.6 (0.77–2.96)	0 (0–16.31)	0 (0–14.76)	1.4 (0.71–2.73)
*B. afzelii* + *B. venatorum*	0.2 (0.01–1.07)	0 (0–16.31)	0 (0–14.76)	0.2 (0.01–0.98)
*B. afzelii* + *B. microti*	0.2 (0.01–1.07)	0 (0–16.31)	0 (0–14.76)	0.2 (0.01–0.98)
*B. afzelii* + *R. helvetica*	1.0 (0.42–2.29)	0 (0–16.31)	0 (0–14.76)	1.0 (0.38–2.11)
*B. spielmanii* + *R. helvetica*	0.3 (0.01–1.33)	0 (0–16.31)	0 (0–14.76)	0.3 (0.01–1.23)
*B. valaisiana* + *R. helvetica*	0.5 (0.10–1.58)	0 (0–16.31)	0 (0–14.76)	0.5 (0.09–1.46)
*B. miyamotoi* + *R. helvetica*	0.2 (0.01–1.07)	0 (0–16.31)	0 (0–14.76)	0.2 (0.01–0.98)
*A. phagocytophilum* + *R. helvetica*	1.2 (0.53–2.52)	0 (0–16.31)	0 (0–14.76)	1.1 (0.49–2.32)
*A. phagocytophilum* + *B. capreoli*	0.2 (0.01–1.07)	0 (0–16.31)	0 (0–14.76)	0.2 (0.01–0.98)
*B. microti* + *R. helvetica*	0.5 (0.10–1.58)	0 (0–16.31)	0 (0–14.76)	0.5 (0.09–1.46)
*B. canis* + *R. raoultii*	0 (0–0.79)	0 (0–16.31)	7.4 (0.96–24.47)	0.3 (0.01–1.23)
*B. canis* + *R. monacensis*	0.2 (0.01–1.07)	0 (0–16.31)	0 (0–14.76)	0.2 (0.01–0.98)
*B. sp. venatorum* + *R. helvetica*	0.3 (0.01–1.33)	0 (0–16.31)	0 (0–14.76)	0.3 (0.01–1.23)
*B. spielmanii* + *B. valaisiana* + *R. helvetica*	0.7 (0.2–1.82)	0 (0–16.31)	0 (0–14.76)	0.6 (0.18–1.68)
*B. afzelii* + *A. phagocytophilum* + *R. helvetica*	0.2 (0.01–1.07)	0 (0–16.31)	0 (0–14.76)	0.2 (0.01–0.98)
*B. garinii* + *A. phagocytophilum* + *R. helvetica*	0.2 (0.01–1.07)	0 (0–16.31)	0 (0–14.76)	0.2 (0.01–0.98)
*B. afzelii* + *B. microti* + *R. helvetica*	0.2 (0.01–1.07)	0 (0–16.31)	0 (0–14.76)	0.2 (0.01–0.98)
Coinfections TOTAL	7.9 (5.97–10.42)	0 (0–16.31)	7.4 (0.96–24.47)	7.6 (5.76–9.94)

## Discussion

**Fig. 2:**
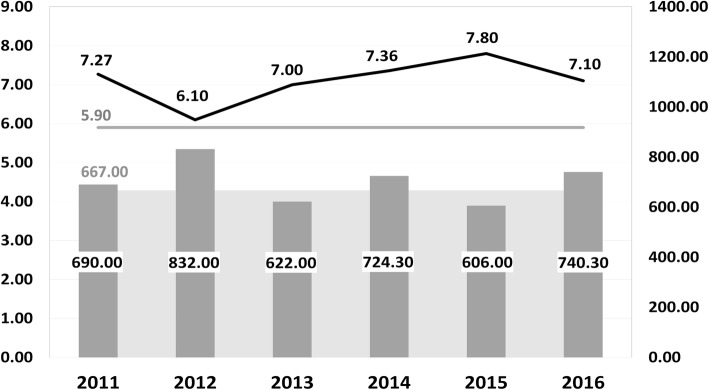
Annual air temperature and precipitation trends in Latvia, 2011–2016. Data source: Latvian Environment, Geology and Meteorology Centre, https://www.meteo.lv/en/. Gray area: the usual average annual precipitation; dark gray columns: the mean annual precipitation; gray line: the usual average annual air temperature; black line: the mean annual air temperature

Apart from the concerns about human health, there is an increasing interest in monitoring of canine tick-borne diseases, and how the eco-epidemiological patterns of the spread of ticks and pathogens change through time. This study aimed to investigate the prevalence of the pathogen species in ticks removed from dogs in Latvia and to analyze any possible changes between years 2011 and 2016. The results showed that, in our settings, monitoring at time points six years apart was not sufficient to observe any significant changes in the prevalence rate of the tick-borne pathogens, which have been circulating in the environment for a long time, and/or for already established loci. These results indicate the relative stability of the environmental processes. Similar results were obtained in a French study where no evidence was observed for a climate-associated increase in infection risk over the 7-year period [14]. However, during the last decade, the spread of *D. reticulatus* has been notable in several European countries, including Latvia and neighboring Lithuania [12, 15]. In this study, almost 7% of ticks removed from dogs in 2016 were *D. reticulatus* comparing to none in 2011, and the difference was statistically significant. This result is in accordance with previous observations: while no *Dermacentor* ticks were collected by flagging in years 2005–2007 in Latvian regions, new localities with *D. reticulatus* occurrence have been found in southern Latvia in the years 2013–2014 [12, 16]. Moreover, our study demonstrated that, until the year 2016, the spreading of *D. reticulatus* ticks occurred further to the north than previously reported - up to the Gulf of Riga (Fig. 1). Climate is probably the major driver to the presence or absence of a tick species in a given territory [17]. Indeed, the mean annual air temperature in Latvia in years 2011–2016 was 0.2–1.9 °C higher than the usual average + 5.9 °C, while the mean annual precipitation fluctuated (Fig. 2; Data source: Latvian Environment, Geology and Meteorology Centre, https://www.meteo.lv/en/). Importantly, the mean air temperature in the Baltic States including Latvia is increasing fastest in winter and spring, and decrease in the snow cover duration during the 1961–2015 period. In addition, a change of the type of winters after 1989 with a later snow cover formation and earlier snowmelt was observed for this area [18]. Thus, while various biotic and abiotic variables influencing overall *D. reticulatus* tick abundance have been reported [19], it could be proposed that climate change supported the emergence of *D. reticulatus* foci in Latvia.

Further, 13 tick-borne human and canine pathogens were detected in ticks removed from dogs this study. In total, 35.8 and 40.0% of ticks were pathogen-positive in 2011 and 2016, respectively; this difference was not statistically significant. The spectrum of detected pathogens was compatible to those reported recently in dog-associated ticks collected in Germany, Italy, Belgium and Poland [20–23].

The most prevalent pathogen genus was *Rickettsia*, it was detected in 24.3% of *I. ricinus*, 8.3% of I*. persulcatus* and 11.1% of *D. reticulatus* ticks. Interestingly, in studies of pet-associated ticks, *Rickettsia* spp. was detected in 18.4 and 14.1% of *Ixodes* ticks in Italy and Belgium, respectively, while over 50% of *I. ricinus* were positive for *Rickettsia* spp. in Poland and Germany; also, *Dermacentor* ticks were pathogen-negative in Belgium, but 39% of *D. reticulatus* were found to be *Rickettsia*–positive in the study in Germany [20–23]. Such non-uniform prevalence of pathogens could be related to many variables including, among others, existence of natural foci, tick and host abundance, sampling area and sampling season. For example, large differences in pathogen frequencies in questing *Dermacentor* ticks (31.4–78.3%) were observed between sampling sites in Germany [19]. However, methodology-related factors such as the level of tick engorgement, feeding on an infected animal and sensitivity of the methods used should also be considered.

In our study, three *Rickettsia* species were identified: *R. helvetica*, *R. monacensis* and *R. raoultii. R. helvetica* has been isolated from *Ixodes* ticks in many European and Asian countries, however, its pathogenicity is relatively unknown. While there is some evidence that it may cause disease in humans [24], there are no such reports regarding clinical cases in dogs. On the other hand*, R. monacensis* is recognized as an emerging human pathogen, as cases of infection in humans were reported in Spain, Italy, the Netherlands and South Korea [25]. *R. monacensis* was also identified in a blood sample of a dog (0.7%) in Maio Island, Cape Verde archipelago, however, its pathogenicity in animals is still unknown [26]. *R. raoultii* is frequently detected in multiple tick species and, along with *R. slovaca*, is a causative agent of a syndrome in humans known as DEBONEL/TIBOLA (*Dermacentor*-borne necrosis erythema and lymphadenopathy/Tick-borne lymphadenopathy) [27]. It is a newly recognized emerging disease, as its incidence has been increasing in Europe during the last decade [28]. Clinical cases in animals induced by *R. raoultii* have not been described so far, however, in dogs in Germany *R. raoultii* DNA was detected in 0.68% of samples and a seroprevalence of 2.8% was reported [29, 30].

*A. phagocytophilum* is the etiologic agent for Human Granulocytic Anaplasmosis (HGA), which occurs in America, Europe and Asia [31]. There are reports on granulocytic anaplasmosis in a variety of domestic and wild animal species including dogs, cats, horses and cattle [32]. The main vector in Europe is *I. ricinus*, however, *A. phagocytophilum* has been detected in questing ticks of many species including *D. reticulatus* and *I. persulcatus*, and the overall infection rate of *I.ricinus* ticks in Europe varied from 0.4% to even 33.9% in some localities (reviewed in [32]). Seroprevalence studies in European dogs indicated that 3 to 57% of dogs carried *A. phagocytophilum* [33], while in Latvia, *A. phagocytophilum* seroprevalence in dogs was significantly higher in the *I. ricinus* region than in the *I. persulcatus* region (12.5% vs 2%) [34]. In the present study, 6% of *I. ricinus* ticks removed from dogs were *A. phagocytophilum*-positive, confirming risks present for humans and animals.

In *I. ricinus*, a member of the relapsing fever group spirochete *B. miyamotoi*, as well as Lyme-disease borrelia *B. afzelii*, *B. garinii*, *B. valaisiana*, *B. spielmanii* were present, but no *Borrelia*-positive *D. reticulatus* ticks were detected in our study. While these pathogens are of a high importance to human health, studies in Europe have shown that many dogs are exposed to *Borrelia*, but only a small number of seropositive animals ever have a clinical disease (reviewed in [35]). According to the American College of Veterinary Internal Medicine consensus update on Lyme borreliosis in dogs and cats it is stated that in dogs residing in North America Lyme borreliosis is associated only with *B. burgdorferi* sensu stricto, and it has not yet been proven that borrelia found in Europe can cause clinical signs in dogs [36]. Nevertheless, for the future reference and from a standpoint of the OneHealth perspective it is important to find out, which borrelia species are prevalent in ticks removed from animals.

Among *Babesia*, *B. microti*, *B. venatorum*, *B. capreoli* and *B. canis* were detected in this study. Both *B. microti* and *B. venatorum* are considered to pose a zoonotic risk to humans, but no reports exist that they either infect or cause disease in dogs. On the other hand, 1.6% of ticks carried *B. canis*, the agent of canine babesiosis. Importantly, none *B. canis*-positive field-collected ticks were detected in Latvia in the 2005–2007 time frame, while the first autochthonous canine babesiosis cases in the country were reported between years 2009 and 2011, and *B. canis* was detected in *D. reticulatus* ticks in Latvia in the 2013–2015 time frame [11, 16, 37]. Unsurprisingly, in this study, the prevalence of *B. canis* in *D. reticulatus* was significantly higher than in *I. ricinus* ticks (14.8% vs 1%; *P* ≤ 0.05). The presence of *B. canis* in *I. ricinus* observed here is in accordance with the study of Cieniuch and colleagues [38] in Poland, which found out that around 1% of field-collected *I. ricinus* ticks were infected, and may explain the cases of autochthonous canine babesiosis in Latvia in 2009–2011, when *Dermacentor* ticks were apparently absent in the country [11]. However, future studies are needed to confirm the role of this tick species. On the other hand, a possibility of a cross-infection exists, when multiple ticks, including infected *D. reticulatus*, are feeding on one animal, or if the dog itself is a carrier of the disease.

While most people in Latvia are aware of human tick-borne diseases, information to the general public on the same topic in house-hold pets is insufficient in Latvia, leading to common misconceptions such “my dog/cat is exposed to ticks so frequently that it is immune to them” (Seleznova M., personal communication). This lack of awareness also means that a lot of pet owners disregard regular anti-tick treatments to their animals during the active season as unnecessary. This situation was exacerbated during the last decade because of the speedy spreading of *D. reticulatus*, which were previously absent in the country, and the risk of infection arising from this tick species populations is not sufficiently investigated. The results of this study confirmed that the spread of novel vectors could bring additional risk of exposure to novel emerging pathogens to pets and their owners, as both *B. canis* and *R. raoultii* were shown to be highly associated with *D. reticulatus* ticks. In addition, the clear possibility of dog’s infestation with several ticks in the sympatric areas for *Ixodes* and *Dermacentor* tick species increases the probability of the co-infection with several pathogens, which, in turn, could increase the risk of severe pathology, and complicates both, diagnosis and therapy. Infection with tick-borne pathogens can also be complicated by other arthropod-borne diseases that share the tick biohabitat, and co-infection could partially explain variations in clinical presentation, pathogenicity and response to therapy in dogs [10].

In this study, a co-infection with two and three tick-borne pathogens was detected in 7.9 and 7.4% of *I. ricinus* and *D. reticulatus* samples, respectively, and a high variability of co-infections was observed. In total, 19 different pathogen combinations were detected among the samples, none of which appeared to be a dominant pattern. This result indicates that a substantial risk of the co-infection with multiple tick-borne pathogens exists, and the combination of pathogens appears to be a dynamic process which varies depending on the changes in the prevalence of separate pathogens in nature. Thus the awareness regarding possible co-infections in ticks should be increased and further studies are needed, especially against the background of the climate change, the emergence and the spread of the sympatric areas for *Ixodes* and *Dermacentor* tick species, and increasing importance of pet travel.

Several limitations of this study should be outlined. First, only adult ticks were included in this study. While the removal of the adult ticks from animals is relatively easy, the molecular analysis of nymphs could provide additional important data. Secondly, the ticks were collected from dogs and were partially or fully engorged. Thus, a possibility exists that some tick samples were pathogen-positive because of feeding on a positive dog. Also, the number of *I. persulcatus* ticks available for this study was very low and did not mirror the actual spread of this tick species in Latvia (according to data presented in [16, 34, 39]. Only *R. helvetica* was identified in these samples, however, the presence of different pathogens in the field-collected *I. persulcatus* ticks such as *Babesia*, *Borrelia* and *Ehrlichia* have been shown previously in Latvia.

## Conclusions

In conclusion, the results of this study clearly demonstrate the potential danger from the inadvertent introduction of novel disease pathogens and vectors in Latvia. A substantial risk of the co-infection with multiple tick-borne pathogens in dogs exists, and the combination of pathogens appears to be a dynamic process which varies depending on the changes in the prevalence of separate pathogens in nature. Further evaluation of the role of *D. reticulatus* as a vector of pathogens to humans and animals is required, and awareness of tick-borne disease caused by *R. raoultii* and *B. canis* needs to be increased in human and veterinary medicine.

## Methods

### Samples collection

Tick samples were collected from dogs in different regions of Latvia in years 2011 and 2016. Only adult ticks were included in this study. We calculated the intensity of the infestation as the mean number of ticks per infested host.

Ticks were removed by veterinarians during the routine visit, preserved in 70% ethanol and stored individually at − 20 °C after morphological identification [40].

### DNA isolation

Ticks were washed with 70% ethanol, dried, transferred into individual tubes and crushed in 300 μl of sterile water. 50 μl of digestion buffer [30 mM Tris-HCl (pH 8.0), 75 mM EDTA (pH 8.0), 0.3 M NaCl, 1.5% SDS] and 2.5 μl of proteinase K (20 mg/ml) were added to 100 μl of tick suspension, and the mixture was incubated at 50 °C for 1 h. DNA was extracted by phenol/chloroform method and stored at − 20 °C.

### Differentiation of *Ixodes* tick species

All *Ixodes* tick samples were analyzed by using real-time PCR method as described elsewhere [41].

### Detection of tick-borne pathogens

All samples were tested for the presence of *Babesia* sp., *Borrelia* sp., *A. phagocytophilum* and *Rickettsia* sp. using nested polymerase chain reaction (PCR) that targeted *18S rRNA*, *16S rRNA*, *16S rRNA* and *gltA* gene, respectively. All primers are listed in Table 4. The PCR reagents were purchased from Thermo Fisher Scientific, USA. All PCR reactions were performed in a final volume of 26 μl, containing 1x Taq Buffer with (NH_4_)_2_SO_4_, 2.5 mM MgCl_2_, 100 μM of each dNTPs, 0.2 μM of each primer, 0.8 U of Taq DNA polymerase (recombinant) and 2 μl of DNA template. Further, 2 μl of the PCR product from the first reaction was used as a template for nested PCR.

**Table 4:** Primers used in this study

Pathogen/target gene	Primer	Sequence (5′- 3′)	Amplicon size, bp ^a^	Reference
*Borrelia* spp./ *16S rRNA*	16S1A	CTAACGCTGGCAGTGCGTCTTAAGC	724	[42]
	16S1B	AGCGTCAGTCTTGACCCAGAAGTTC		[42]
	16S2A	AGTCAAACGGGATGTAGCAATACA	657	[42]
	16S2B	GGTATTCTTTCTGATATCAACAG		[42]
*A. phagocytophilum*/ *16S rRNA*	ge3a	CACATGCAAGTCGAACGGATTATTC	932	[43]
	ge10r	TTCCGTTAAGAAGGATCTAATCTCC		[43]
	ge9f	AACGGATTATTCTTTATAGCTTGCT	546	[43]
	ge2	GGCAGTATTAAAAGCAGCTCCAG		[43]
*Rickettsia* sp./ *gltA*	VC29	GCGGAAGCCGATTGCTTTAC	1108–1111	This study
	CS-1069	GAGGGTCTTCGTGCATTTCTT		[44]
	RH314	AAACAGGTTGCTCATCATTC	898–901	[45]
	CS-1069	GAGGGTCTTCGTGCATTTCTT		[44]
*Babesia* spp./ *18S rRNA*	5-22F	GTTGATCCTGCCAGTAGT	1622–1731	[46]
	1661R	AACCTTGTTACGACTTCTC		[46]
	455-479F	GTCTTGTAATTGGAATGATGGTGAC	310–368	[46]
	793-772R	ATGCCCCCAACCGTTCCTATTA		[46]

^a^ Amplicon size depends on the pathogen genospecies, *bp* base pairs

The PCR assays for *Babesia* sp. detection were performed under the following conditions: an initial denaturation at 95 °C for 3 min; 35 cycles of denaturation at 95 °C for 20 s, primer annealing at 55 °C for 30 s, and elongation at 72 °C for 1 min; and a final elongation step at 72 °C for 5 min. Nested PCR assays were performed under the same conditions, but with reduced time (30 s) for the elongation step. This assay was *Babesia-*and *Theileria*-specific. The PCR assays for *Borrelia* sp. detection were performed under the following conditions: an initial denaturation at 95 °C for 3 min; 30 cycles of denaturation at 95 °C for 20 s, primer annealing at 63 °C for 20 s, and elongation at 72 °C for 40 s; and a final elongation step at 72 °C for 5 min. Nested PCR assays were performed under the following conditions: an initial denaturation at 95 °C for 3 min; 35 cycles of denaturation at 95 °C for 20 s, primer annealing at 56 °C for 20 s, and elongation at 72 °C for 30 s; and a final elongation step at 72 °C for 5 min. The PCR assays for *A. phagocytophilum* detection were performed under the following conditions: an initial denaturation at 95 °C for 3 min; 35 cycles of denaturation at 95 °C for 20 s, primer annealing at 58 °C for 20 s, and elongation at 72 °C for 40 s; and a final elongation step at 72 °C for 5 min. Nested PCR assays were performed under the same conditions, but reduced to 30 cycles and a reduced time (30 s) in the elongation step was used. The PCR assays for *Rickettsia* sp. detection were performed under the following conditions: an initial denaturation at 95 °C for 3 min; 35 cycles of denaturation at 95 °C for 20 s, primer annealing at 55 °C for 30 s, and elongation at 72 °C for 90 s; and a final elongation step at 72 °C for 5 min. Nested PCR assays were performed under the same conditions, but a reduced time (20 s) in the denaturation step was used. As positive controls, plasmids with inserts derived from *B. canis*, *B. afzelii* ACAI strain, *A. phagocytophilum* Webster strain and *R. raoultii* were used, respectively. Positive and negative controls were included in all PCR amplification.

Amplicons were purified and analyzed by Sanger sequencing on both DNA strands using an ABI Prism 3100 Genetic Analyzer (PerkinElmer, Waltham, MA, USA). Pathogens were identified using the GenBank database. Pathogen genotypes were assigned based on sequence similarity (99–100%) to the corresponding gene of the reference strains (GenBank accession numbers: *B. afzelii* NR_104748.1; *B. garinii* NR_043413.1; *B. spielmanii* NR_104871.1; *B. valaisiana* NR_036807.1; *B. miyamotoi* NR_025861.1; *B. microti* XR_001160977.2; *B. canis* AY072926.1; *B. venatorum* AY046575.1; *B. capreoli* FJ944827.1; *R. raoultii* NZ_CP019435.1; *R. helvetica* NZ_CM001467.1; *R. monacensis* NZ_LN794217.1. All primers and probes were synthesized by Metabion International AG, Germany, and all PCR reagents were purchased from ThermoScientific, Waltham, MA, USA.

### Statistical analysis

All ticks were processed individually, and the prevalence was expressed as a percentage. A *P* value was calculated using the two-sided Fisher’s exact test (GraphPad Prism 6, GraphPad Software, La Jolla, CA, USA). Values of *P* ≤ 0.05 were considered significant. The mean intensities of tick infestation of dogs between years were compared by the Mann-Whitney U test (MedCalc Software, Version 19.1). Prevalence of pathogens was calculated with 95% confidence intervals of a proportion by the “exact” method of Clopper and Pearson (GraphPad Prism 6). *P* values were adjusted for the multiple testing by Holm correction in R using the R Statistical Package.

## Data Availability

The datasets used and/or analysed during the current study are available from the corresponding author on reasonable request.

## Abbreviations

dNTP: Deoxyribonucleotide triphosphate
EDTA: Ethylenediaminetetraacetic acid
PCR: Polymerase chain reaction
SDS: Sodium dodecyl sulfate
